# Islet-on-a-chip for the study of pancreatic β-cell function

**DOI:** 10.1007/s44164-021-00005-6

**Published:** 2021-12-02

**Authors:** Júlia Rodríguez-Comas, Javier Ramón-Azcón

**Affiliations:** 1 Biosensors for Bioengineering Group, Institute for Bioengineering of Catalonia (IBEC), The Barcelona Institute of Science and Technology (BIST), Baldiri i Reixac, 10-12, 08028 Barcelona, Spain; 2 ICREA-Institució Catalana de Recerca i Estudis Avançats, 08010 Barcelona, Spain

**Keywords:** Diabetes, Pancreatic islets, Organ-on-chip, Islet-on-chip, Microfluidics

## Abstract

Diabetes mellitus is a significant public health problem worldwide. It encompasses a group of chronic disorders characterized by hyperglycemia, resulting from pancreatic islet dysfunction or as a consequence of insulin-producing β-cell death. Organ-on-a-chip platforms have emerged as technological systems combining cell biology, engineering, and biomaterial technological advances with microfluidics to recapitulate a specific organ’s physiological or pathophysiological environment. These devices offer a novel model for the screening of pharmaceutical agents and to study a particular disease. In the field of diabetes, a variety of microfluidic devices have been introduced to recreate native islet microenvironments and to understand pancreatic β-cell kinetics in vitro. This kind of platforms has been shown fundamental for the study of the islet function and to assess the quality of these islets for subsequent in vivo transplantation. However, islet physiological systems are still limited compared to other organs and tissues, evidencing the difficulty to study this “organ” and the need for further technological advances. In this review, we summarize the current state of islet-on-a-chip platforms that have been developed so far. We recapitulate the most relevant studies involving pancreatic islets and microfluidics, focusing on the molecular and cellular-scale activities that underlie pancreatic β-cell function.

## Introduction

### Type 2 diabetes: a global pandemic

The maintenance of glucose homeostasis is critical for normal body function. Despite wide daily fluctuations in glucose levels, glucose concentrations need to be maintained within a narrow range to avoid harmful effects of hyperglycemia and to prevent hypoglycemia. This tight regulation is accomplished by the coordinated actions of different organs and tissues, among which the pancreas represents a key player by secreting the blood sugar-lowering insulin hormone by pancreatic β-cells and its opponent glucagon by α-cells.

Diabetes mellitus (DM) is a group of chronic metabolic disorders all characterized by hyperglycemia. It occurs either when the pancreatic islets do not produce enough insulin or when the peripheral tissues cannot efficiently use the insulin produced by the pancreas. Persistent high blood glucose levels cause generalized vascular damage affecting the kidneys (nephropathy) [1], the eyes (retinopathy) [2], the nerves (neuropathy) [3], and the heart (cardiovascular disease) [4], leading to disabilities and premature death. According to the World Health Organization (WHO), diabetes is among the top 10 causes of death globally. The prevalence of diabetes has been increasing over the recent decades. Indeed, the International Diabetes Federation (IDF) reported that in 2019 there were 463 million people between 20 and 79 years worldwide with diabetes, accounting for the 9.3% of the global adult population. If this trend continues, it is expected to increase to 700 million by 2045. The economic burden of diabetes has a high impact on the global healthcare system and the global economy as it has been estimated that the direct annual cost of diabetes worldwide is more than USD 760 billion [5]. This data evidences the need for more clinical and preventive intervention programs and highlights the importance of carrying out more basic research in order to understand the underlying mechanisms of the disease.

According to the etiology of the disease, the American Diabetes Association (ADA) classifies diabetes into the following general categories [6]:

1. Type 1 diabetes (T1D). Autoimmune disease in which β-cell destruction usually leads to absolute insulin deficiency. It accounts for 5–10% of all diabetes [7, 8].
2. Type 2 diabetes (T2D). Progressive loss of β-cell insulin action and/or secretion, frequently on the background of insulin resistance. T2D represents more than 90% of all cases worldwide [9].
3. Gestational diabetes mellitus (GDM). Diabetes with onset or diagnosed during pregnancy that was not clearly overt diabetes prior to gestation. The prevalence of gestational diabetes varies from ~2 to ~12% depending on the different sets of criteria around the world [6, 10].
4. Other types of diabetes comprise monogenic diabetes syndromes (such as neonatal diabetes and maturity-onset of the young [MODY]), diseases of the exocrine pancreas (such as cystic fibrosis and pancreatitis), and drug- or chemical-induced diabetes (such as with glucocorticoids use in the treatment of the human immunodeficiency virus (HIV) or after organ transplantation) [6].

Type 2 diabetes (T2D) is the most common type of diabetes and represents more than 90% of all diabetes cases worldwide. Therefore, it will be presented in detail in this review. It is characterized by a combination of a decreased response to insulin action in target tissues (e.g., muscle or adipose tissue) and impaired insulin secretion from pancreatic β-cells in response to secretory stimuli [9], the latter being the ultimate cause that leads to the development and progression of T2D.

### The main players: the islets of Langerhans

**Fig. 1:**
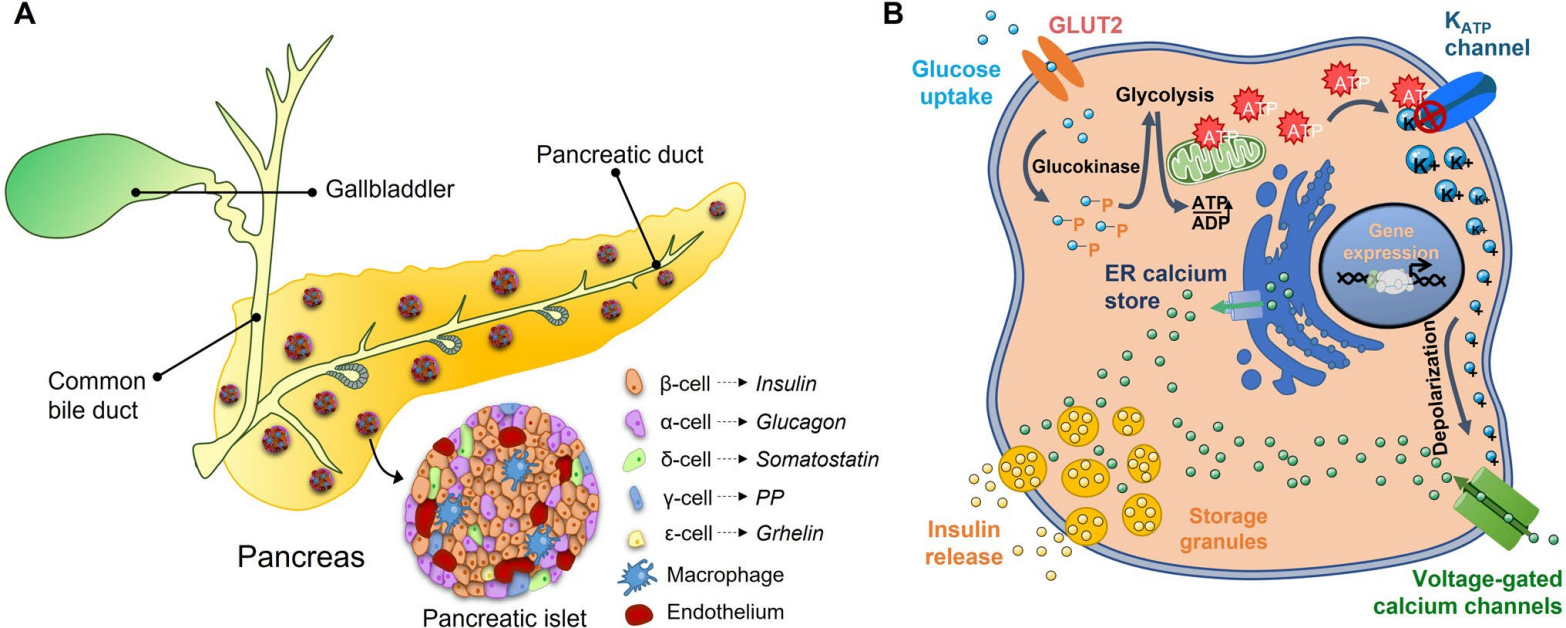
**A** Schematic representation of the pancreas and the pancreatic islet. Islets are composed of 5 endocrine cells; β-cells, α-cells, δ-cells, γ-cells, and ε-cells which secrete different hormones into the bloodstream. Non-endocrine cells such as macrophages and endothelial cells are also present in the islets of Langerhans. **B** Glucose uptake and insulin release from a β-cell. At basal levels of blood glucose, the K_ATP_ channel remains open, maintaining membrane hyperpolarization, Ca2+ channel closure, and inhibiting insulin secretion. A rise in blood glucose drives oxidative phosphorylation and ATP production, resulting in the closure of KATP channels, plasma membrane depolarization, calcium influx, and insulin vesicle exocytosis

The pancreas acts as an exocrine and endocrine organ, being a key player in the regulation of macronutrient digestion and metabolism homeostasis by releasing a variety of digestive enzymes and pancreatic hormones. Over 98% of the pancreas consists of acinar or exocrine cells. As an exocrine gland, it produces several digestive enzymes, such as amylase, pancreatic lipase and trypsinogen, and bicarbonate that are secreted into the duodenum via the pancreatic duct [11]. In contrast, as an endocrine gland, the pancreas secretes various hormones that regulate blood glucose levels. The endocrine cells are clustered together, forming the so-called islets of Langerhans. Pancreatic islets are small, round-shaped structures scattered throughout the exocrine pancreatic tissue that accounts for only 1–2% of the entire organ [12]. They are composed of 5 different endocrine cell types, including glucagon-secreting α-cells, which represent 20–30% of the total islet cells; amylin or islet amyloid polypeptide (IAPP), C-peptide, and insulin-secreting β-cells, which account for 60–70% of the total cells; somatostatin-secreting δ-cells, which constitute 3–10% of the total cells; pancreatic polypeptide (PP)-secreting γ-cells, which comprise 3–5% of the total islet cells; and ghrelin-secreting ε-cells, which take up 1% of the total islet cells [13, 14] (Fig. 1A).

β-cells are the predominant cell type within the pancreatic islet in mammals and are the unique source of circulating insulin. In rodent islets, the vastly predominating β-cells are clustered in the core of the islet, surrounded by a mantle of α, δ, and γ cells. In human islets, β-cells intermingle with the other cell types throughout the islet [14]. Human islets tend to contain fewer β-cells and more α-cells compared to rodent islets [15]. Adequate and proper β-cell function requires normal β-cell integrity, which is critical for the appropriate response to continuous fluctuating metabolic demand for insulin.

Under physiological conditions, the maintenance of glucose homeostasis with blood glucose levels within a narrow physiological range relies on the regulation of insulin secretion through nutrient availability, hormones, and neural inputs. Among these factors, glucose is the most important physiological secretagogue for insulin, regulating β-cell function through coordinated stimulation of insulin gene transcription, proinsulin biosynthesis, and insulin secretion from β-cells. Thus, the β-cell is dedicated to convert daily fluctuations in blood glucose concentrations during the postprandial and postabsorptive phases (typically from 4.5 to 8 mM in humans) into large changes in insulin secretion within minutes. Therefore, β-cells are “fuel-sensors” that constantly monitor and respond to dietary nutrients in order to best meet the requirements of the organism by synthesizing and secreting proper amounts of insulin [16].

Glucose enters the β-cells via facilitative glucose transporter 2 (GLUT2) in rodents (GLUT1 in humans) [17] and is rapidly phosphorylated by glucokinase and converted to pyruvate by the glycolytic pathway. Pyruvate oxidation in the mitochondria through the tricarboxylic acid cycle leads to a series of reactions that generate NADH from NAD+ (nicotinamide adenine dinucleotide). Of note, unlike many other cell types, mature β-cells express very low levels of lactate dehydrogenase (LDH) and do not regenerate NAD^+^ via lactate formation. Alternatively, β-cells possess high activity of two NADH shuttles, the glycerol-3-phospate and the malate/aspartate shuttle, to transfer NADH into the mitochondrial for oxidative metabolism and ATP (adenosine triphosphate) production. The increase in intracellular ATP/ADP (adenosine triphosphate/adenosine diphosphate) ratio promotes the closure of ATP-sensitive potassium (K_ATP_) channels, depolarization of the plasma membrane, and opening of voltage-dependent calcium [(Ca^2+^)] channels. The rise in [Ca^2+^] into the cell is followed by exocytosis of the insulin-containing granules [18] (Fig. 1B).

Insulin is a peptide hormone that orchestrates an integrated anabolic response to nutrient availability. It acts by binding to the insulin receptor on the plasma membrane of target cells [19]. Its synthesis in the β-cells, followed by a quality control, delivery, action, and clearance, is delicately regulated by different organs and tissues. Although many cell types express insulin receptors, the role of insulin in glucose homeostasis is characterized by the direct effects of this peptide on skeletal muscle, liver, and white adipose tissue. Thus, the journey of insulin in the body is an example of a highly coordinated and integrated cellular physiology [20].

**Fig. 2:**
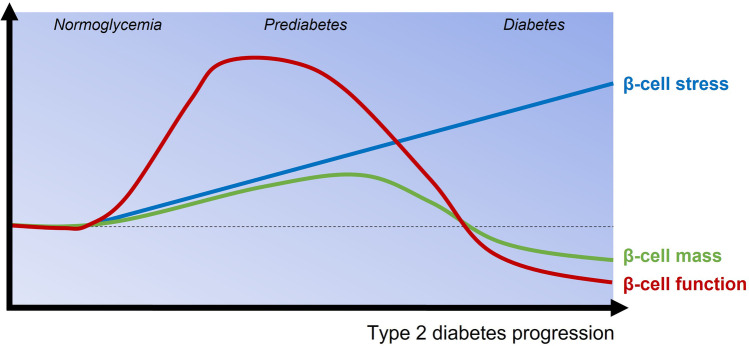
Contribution of the β-cell mass and function and the progression of β-cell stress to the pathogenesis of T2D. In a prediabetic state, usually characterized by glucose intolerance and impaired glucose tolerance, β-cells initially compensate to maintain normoglycemia by increasing their workload. However, this continuous overwork is accompanied with cellular stress and exhaustion. At this point, β-cells are no longer able to cope with the metabolic demands and hyperglycemia manifests. This, together with external stress factors, leads to β-cell dysfunction and loss, finally culminating in overt T2D.

Patients with T2D present a progressive decline in pancreatic β-cell function, with concomitant impairment of insulin secretion. Multiple mechanisms underlie this β-cell dysfunction, including glucolipotoxicity [21–24], oxidative stress [25, 26], endoplasmic reticulum (ER) stress [27–29], and the formation of amyloid deposits in the islets [30–35], all associated with inflammatory responses [36, 37]. All these physiological stressors may impact the β-cell function in the context of metabolic overload and insulin resistance commonly found in human obesity-linked T2D. In this situation, while β-cells initially respond by activating a compensatory response to maintain normoglycemia, expanding β-cell mass and improving their insulin secretory capacity; eventually, they initiate several pathologic programs that synergistically promote β-cell dysfunction and, ultimately, β-cell death [38] (Fig. 2).

Therefore, exploring the pathologic conditions or β-cell stressors that lead to β-cell dysfunction is necessary to understand and intercede in the disease progression, especially in the early stages of the pathology. For this reason, measurements of insulin secretion dynamics, traditionally assessed by glucose-stimulated insulin secretion (GSIS) assays, are of significant clinical relevance.

## Organ-on-a-chip technology

The study of insulin secretion aimed at addressing islet functionality requires monitoring insulin release over time. However, the existing standard assays for islet functionality and viability present limited physiological relevance: usually, it involves numerous animals to assess the capacity of the pancreas to secrete insulin after a glucose challenge and/or static assay incubations of isolated islets subjected to low and high glucose concentrations in order to evaluate the insulin stimulation index. These experiments are laborious, require long processing time, and are usually followed by off-line quantification of insulin by an enzyme-linked immunosorbent assays (ELISA) or a radioimmunoassay (RIA) [39]. Hence, its readout is not obtained in real-time, making it impossible to measure rapid spatiotemporal responses. Additionally, these measurements do not have the sufficient sensitivity to measure insulin released by a single islet, and consequently these measurements are usually performed using batches of islets.

Harvesting pancreatic islets from rodents or human donors is difficult. For this reason, minimizing the number of islets required for experiments is fundamental. Moreover, the release of hormones from pancreatic islets occurs within minutes in a pulsatile fashion in response to an appropriate stimulus. Thus, to resolve the secretion dynamics, methods able to quantify insulin secretion with high temporal resolution are required.

**Fig. 3:**
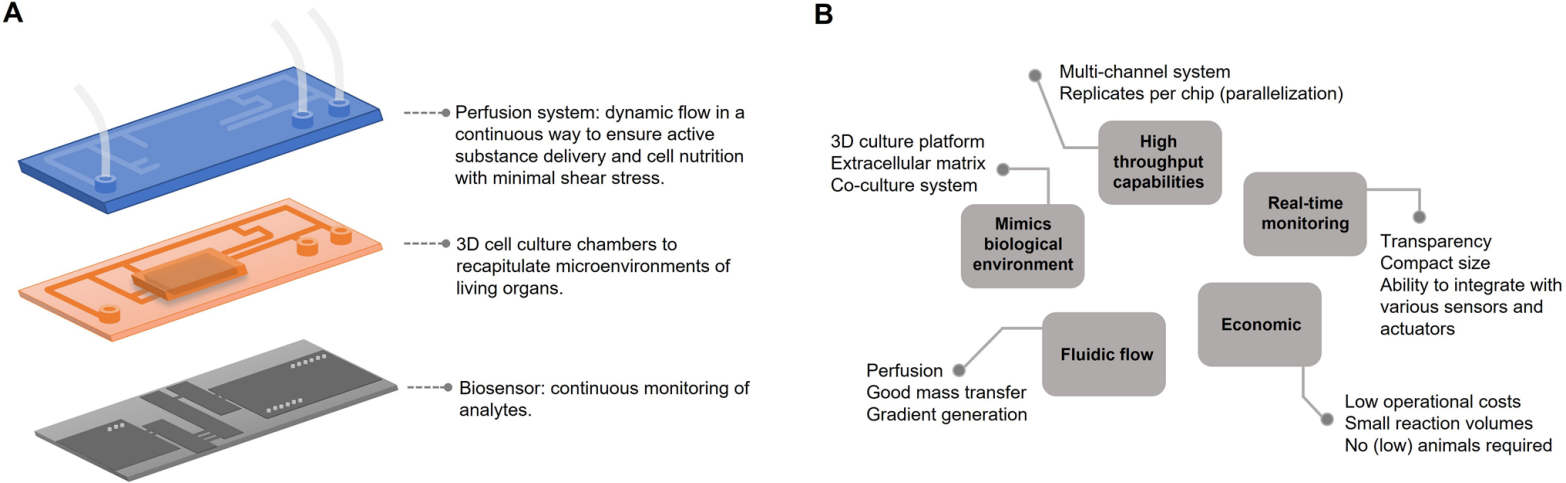
**A** Microfluidic chip and biosensing. The development of microfluidic systems aims to recapitulate in vivo cellular model by integrating a perfusion system made by interconnected micro-channels, a 3D cell culture chamber, and a biosensing chamber. **B** Advantages of organ-on-chip systems.

New high-technological advances have allowed the development of microfluidic organ-on-a-chip (OOC) systems to recapitulate in vivo cellular models with a high level of control. Recently, they have been defined as “microfabricated cell culture devices designed to model the functional units of human organs *in vitro*” [40]. These new technological devices are emerging as a powerful tool for the study of multifactorial pathologies such as diabetes. Organ-on-a-chips are usually based on polymeric platforms where living microtissues can be cultured and, in combination with microfluidics, mimic specific functions of one or multiple organs (Fig. 3A). The most commonly used material is polydimethylsiloxane (PDMS), a silicon-based organic polymer with high biocompatibility characteristics, optical transparency to facilitate imaging applications, and ease of assembly [41, 42].

Numerous easy-to-use compatible micro-scale OOC have been developed modeling different tissues (muscle [43], blood vessels [44], lung [45], gut [46], etc.). Advances in OOC fabrication procedures have also allowed the fabrication of multi-organ-on-a-chip, connecting separated compartments together for monitoring complex interactions between multiple organs in a physiological manner [47–49].

The use of organ-on-a-chip devices offers several distinct benefits over conventional techniques for the study of different pathologies (Fig. 3B). The main advantages of these microfluidic systems are (a) the high degree of control over the spatiotemporal organization of in vivo-like tissue architectures, with the potentiality to maintain an optimal microenvironment for the cells; (b) the possibility of performing experiments using less volume of samples, chemicals, and reagents; (c) the capability to precisely control the amount, duration, and intensity of a particular stimulus; and (d) the ability of monitoring in real-time the effects of external cues on cell, tissue, or organ functions. In the context of diabetes research and the study of pancreatic β-cells, specialized cell types which continually monitor and respond to dynamic metabolic fluctuations, this rapid and real-time analysis is of particular interest. In this sense, the development of islet-on-a-chip (IOC) platforms offering continuous monitoring of insulin-producing cells may enable broad implications in understanding the underlying molecular mechanisms of the disease. These platforms can contribute to the discovery of potential therapies, and may offer a therapeutic value for transplants.

Over the last decades, a variety of microfluidic devices have been introduced to recreate physiological microenvironments to study the islet function and to assess the quality of these islets for subsequent in vivo transplantation. However, reports describing islet-on-a-chip devices for the physiological analysis of pancreatic islets are still limited compared to other organs or tissues. In this review, we summarize the current state of islet-on-a-chip platforms that have been developed to recapitulate relevant biological native islet physiology for the study of the islet function. We assess whether these advanced in vitro platforms can detect insulin secretion on-line. We evaluate the possibility of using IOC for the analysis of a single islet as well as for the study of long-term islet experiments. And finally, we provide an overview of the technological advances in research on tissue crosstalk by combining pancreatic islets with other cell types through their integration into microphysiological environments.

### Islet-on-a-chip: a platform design for the study of the β-cell function

Islet β-cell failure is progressive, particularly after hyperglycemia is established, which leads to a decrease in β-cell functionality, dedifferentiation, and ultimately β-cell death. Therefore, identifying those factors that make islets susceptible to dysfunction and failure is critical to understand islet physiology. However, mechanistic studies of the disease are often hindered by static analysis of the islets that cannot provide the details on temporal dynamics of insulin secretion. Here, we offer a comprehensive review of relevant microfluidic devices that have been developed to address some of these limitations, demonstrating different advantages over conventional off-line techniques to elucidate the mechanisms beneath the islet physiology.

One important requirement for the success of these devices is the suitable design of the system for the study of intact pancreatic islets. Adequate and proper β-cell function requires normal β-cell integrity, which is critical for the appropriate response to continuous and fluctuating metabolic demands for insulin. Pancreatic islets present a unique round-shaped cytoarchitecture that has a high impact on the β-cell function [50]. For this reason, a pertinent design for the immobilization of islets without damaging the islet cytoarchitecture is crucial for the miniaturized perfusion setup. Additionally, the generation of on-chip dynamic environments with the appropriate laminar or turbulent flow also needs to be precisely designed and controlled. This will enable prediction of the nature of the fluid and will allow the performance of unique experiments that are difficult to perform using conventional approaches. One last point to consider when fabricating the microfluidic system is the possibility of obtaining transparent devices that can allow the easy integration of optical detection methods like a biosensing system to evaluate different biochemical and molecular readouts on-line [51].

### Multi-islet screening by IOC technology

**Fig. 4:**
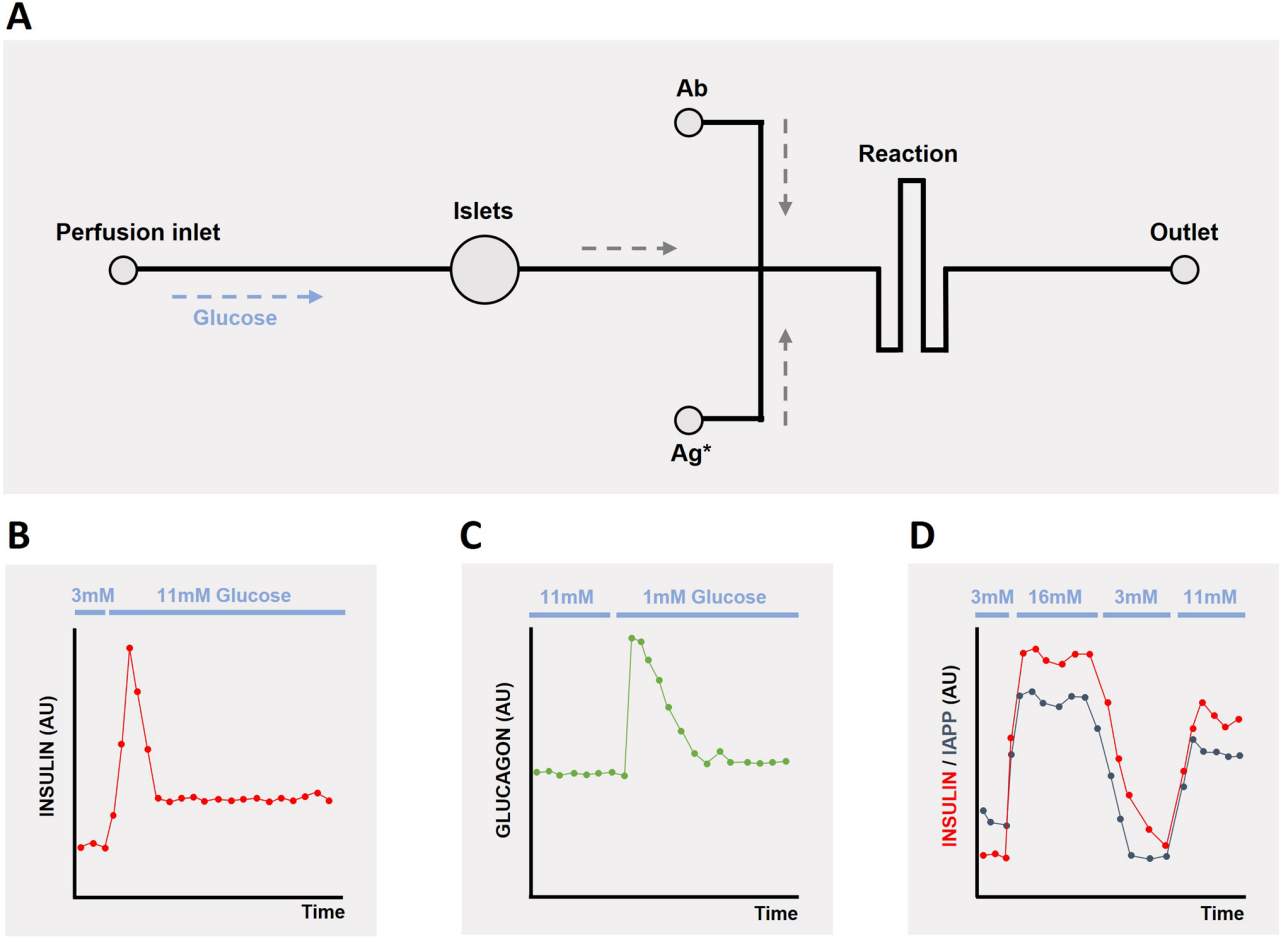
Schematic representation of electrophoresis-based immunoassay microfluidic chips for online detection of hormones released by pancreatic islets. **A** Microfluidic device composed of a perfusion inlet, a chamber to host pancreatic islets, a reaction chamber where hormones react with fluorescent antibodies, and an outlet. Glucose is infused through the perfusion inlet and stimulates the islets, which secrete hormones in response to different glucose concentration. **B**–**D** Different microfluidic devices have been designed for a continuous monitoring of **B** insulin, **C** glucagon, and **D** both insulin and IAPP secretion from islets. Adapted from Reid et al. (2009) [56], Shackman et al. (2012) [57], and Lomasney et al. (2013) [58].

The first studies involving microfluidics and pancreatic islets were performed by the Kennedy group [52–54]. They characterized the development and evaluation of a series of microfluidic devices used for the islet study and illustrated the feasibility of using microfluidics for monitoring β-cell function. These investigations, interrogating a single islet [52, 53], enabled the development of a microfluidic device that incorporated a continuous perfusion and an on-line electrophoresis immunoassay. These first studies were finally completed with the development of a radial microchannel network chip that allowed high-throughput quantitative monitoring of insulin secretion from 4 [54] and 15 [55] islets in parallel. The detection principle was based on on-line immunoassays and established an on-line insulin detection method through mixing of the perfusate with a fluorescent-labeled antibody. Further adjustments allowed the development of a microfluidic electrophoresis chip that was capable of performing serial electrophoretic insulin immunoassays for up to 24h [56]. Following the same procedure, competitive immunoassays on a microfluidic chip were developed to detect and monitor on-line the α-cell secreting hormone, glucagon [57], and to examine the secretory profile of insulin and IAPP (islet amyloid polypeptide) simultaneously [58] (Fig. 4).

Of note, IAPP is a hormone co-secreted by β-cells along with insulin in response to nutrient stimuli [59], and both hormones regulate glucose metabolism in a tightly coordinated manner [60]. Lomasney et al. [58] presented a microfluidic device provided with a two-color detection scheme for on-line measurement of rapid changes in insulin and IAPP in response to glucose through a continuous mixing of fluorescent-labeled antibodies with both peptides secreted by the islet pool and allowing their detection with an inverted fluorescent microscope. Importantly, the human peptide form of IAPP (hIAPP) accumulates, forming amyloid deposits within the pancreas. The aggregation of hIAPP into organized deposits has been considered a pathological characteristic of T2D, contributing to β-cell dysfunction and death and participating in the failure of islet transplantation [61–64]. Therefore, an instrument able to monitor the oscillatory secretion dynamics of this hormone, together with the ability to follow its aggregation pattern over time, could be an extremely valuable tool in diabetes research.

The demands of high-sensitivity analysis of islet function under oscillatory conditions have required more control over experimental parameters. For instance, microfluidic platforms need to offer exquisite capabilities in controlling chemical gradients for understanding β-cell kinetics. In this respect, the design of a dual perfusion network integrated with on-chip staggered herringbone mixers (SHM) to enhance mixing efficiency demonstrated efficacy in establishing high-resolution temporal glucose gradients and allowed optical detection of two separate islet populations [65].

Different functional assays performed in short time periods provided further information regarding the use of microfluidics as an excellent tool to perform comprehensive islet analysis and to obtain a more significant predictive value for islet functionality. In this sense, several microfluidic chips successfully incorporated intracellular fluorescent detection of Ca^2+^ and mitochondrial activity to determine islet cell physiological behavior. As mentioned above, non-diabetic β-cells couple changes in blood glucose in order to secrete the appropriate amount of insulin, and GSIS in the islets is coupled to the metabolic state of β-cells, involving both glycolytic and Krebs cycle metabolism. Mitochondria from β-cells play an essential role in this process, generating factors that couple nutrient metabolism to exocytosis of insulin-containing granules. The release of insulin requires an increase in cytosolic Ca^2+^ levels, which depends on ATP synthesized by the mitochondria. Therefore, the kinetics of insulin secretion is determined by changes of [Ca^2+^] and mitochondrial activity in β-cells. Interestingly, it has been observed that in isolated islets from T2D patients, glucose induces changes in β-cells that have an impact on mitochondrial metabolism, ATP synthesis, and Ca^2+^ oscillations [66, 67]. However, the molecular mechanisms underlying the progressive failure of β-cells are far from being resolved. Limited data on islet metabolism probably resides in the complexity of measuring metabolites while simultaneously stimulating pancreatic islets with oscillatory glucose levels. Microfluidic platforms can take advantage of standard fluorescent labeling methods to allow real-time and non-invasive assessments of metabolic alterations. Indeed, these systems allowed the characterization of the islet functionality by depicting their metabolic activity through fluorescence imagining of calcium influx and mitochondrial membrane potential changes [68, 69], obtaining promising results regarding enhanced spatial-temporal resolution.

The applications of microfluidics for synchronized islet perfusion and fluorescent imaging to simultaneously detect live-cell changes in calcium influx and mitochondrial potentials were the base of a substantial number of devices. However, most of these were usually followed by off-line standard enzyme-linked immunosorbent assay (ELISA) to quantify the amount of secreted insulin [70]. Indeed, the impossibility to detect insulin in real-time has been one of the main limitations of different suitable designs that have been proposed to evaluate islet functionality in response to dynamic glucose stimulation.

Additionally, the integration of glucose and oxygen modulations to IOC platforms has permitted the evaluation of not only the impact of high glucose concentrations but also the effects of hypoxia on the impairment of islet function [71]. The integration of all these desirable features into one single design was recently accomplished, allowing multiparametric measurements of oxygen consumption, NAD(P)H autofluorescence, intracellular Ca^2+^ levels, and off-line detection of insulin [72]. However, the need to fabricate a borosilicate glass device in contrast to using the widely exploited PDMS (polydimethylsiloxane) polymer to minimize the gas permeability and the strong autofluorescence of the polymeric compound makes this device less suitable for widespread use in all laboratories.

The capability of capturing changes in membrane potentials induced by glucose by electrophysiological recordings also provides meaningful integrative readouts closely linked to functionality in β-cells, as membrane potentials of β-cells oscillate in accordance to different glucose concentrations. Indeed, extracellular recording of glucose-induced electrical activity has already been demonstrated with microelectrode arrays (MEAs) on mouse [73] and human pancreatic islets [74]. To provide immediate information of islet activity, a combination of electrophysiology and microelectronics with microfluidics was proposed, attaining a continuous monitoring of electric signals of dissociated islet cells [75] and entire islets [76]. The use of extracellular non-invasively electrodes allowed an automated recording of the cell activity when exposed to different solutions, obtaining a temporal resolution that could not be reached by other approaches. Non-invasive monitoring of pseudo-islets in biomechanical flow conditions has also been achieved with a Raman microspectroscopy. This technique allowed an on-line tracking of the β-cells in response to glucose and enabled visualization of different molecular structures such as lipids, mitochondria, and nuclei [77].

### Interrogating β-cell function at a single islet level

The temporal resolution of many devices when interrogating pancreatic islets has been usually limited by the requirement of working with relatively large volumes of reagents or media and the need to use multiple islets to detect and achieve quantifiable insulin concentrations. Single-islet sensitivity minimizes the number of islets required for testing and enhances the temporal resolution of insulin dynamics. Of interest, pooling islets together entails a decreased temporal resolution in dynamic insulin secretion due to an uncoordinated response of individual islets [18] unless a mechanism for entrainment of pancreatic islets is applied [78]. The advent of microfluidic platforms that precisely rely on a careful design of the microfluidic structures with an accurate control of the flow fostered the integration of several strategies to increase the spatio-temporal control of islets in microfluidics and permitted researchers to resolve the highly dynamic and oscillatory secretion pattern of single islets.

As mentioned above, in 2003, Kennedy and colleagues [52] already presented the first prototype able to interrogate one single islet by a capillary electrophoresis competitive immunoassay. However, the lack of perfusion in the islet chamber was one of the main drawbacks in this first secretion study, resulting in serious limitations in terms of measurements of dynamic insulin secretion and the viability of the islet cells. An advancement over this microfluidic chip, with a continuous perfusion on the islet compartment, was further presented, demonstrating an improvement of the temporal resolution for monitoring insulin secretion while enhancing compatibility with longer-term measurements [53].

**Fig. 5:**
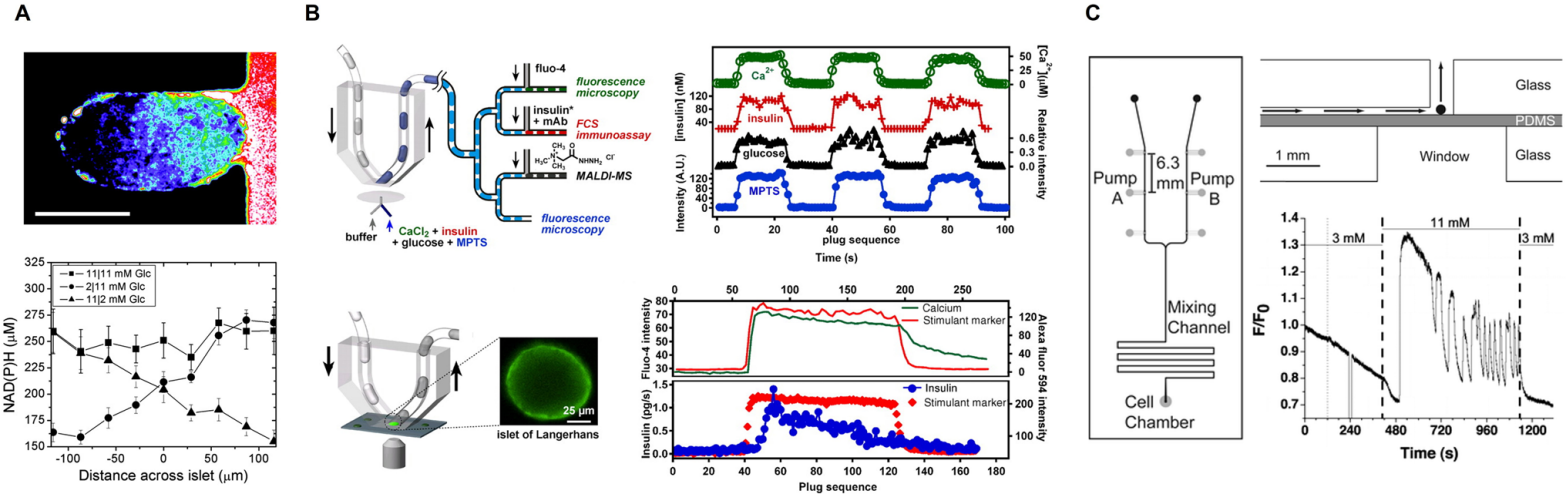
Microfluidic perfusion chips developed for investigating cellular dynamics by measuring intracellular calcium levels and NAD(P)H. **A** Development of a microfluidic device to treat islets with two separate fluid streams. Pancreatic islet after exposure to 2-NBDG (2-(*N*-(7-nitrobenz-2-oxa-1,3-diazol-4-yl)amino)-2-deoxyglucose), a fluorescent glucose analog, in the right stream. This analog is transported into β-cells through GLUT-2 and can be used to track glucose uptake and diffusion directly. A gradient of 2-NBDG intensity was formed across the islet (scale bar, 150 μm). The NAD(P)H concentrations across an islet exposed to 11/11, 2/11, and 11/2 mM glucose on the left/right side. Adapted with permission from Rocheleau et al. (2004) [79]. Copyright © 2004, National Academy of Sciences. **B** Use of a chemistrode to record and analyze pulses of sample solution containing CaCl_2_, insulin, glucose, and a fluorescent dye, MPTS. Left: A schematic of the experiment. An islet was cultured on a glass-bottom dish, and the chemistrode was positioned over the islet. Stimulation and recording took place while the islet was imaged by fluorescence microscopy. A fluorescent image of an islet showing an increase of fluorescence of fluo-4, corresponding to the rise in intracellular [Ca2+]i in the islet upon stimulation. Right: (Upper) Experimental data of Ca2+, insulin, glucose, and MPTS analyses. (Bottom) Graphs showing the [Ca2+]i response and insulin secretion of a stimulated mouse islet. Fluorescence intensity of fluo-4 (green) as an indicator of [Ca2+]i and the intensity of Alexa Fluor 594 (red) as a marker of the stimulant solution. Off-line analysis of plugs collected during recording, showing the fluorescence intensity of Alexa Fluor 594 marker and the calculated insulin secretion rate (blue). Adapted with permission from Chen et al. (2008) [82]. Copyright © 20008, National Academy of Sciences. **C** Top view of the microfluidic perfusion system and side view of the cell chamber. The black sphere represents an islet. The window below the islet facilitated fluorescence monitoring of intracellular Ca2+ levels. Single islet [Ca2+] changes in response to oscillatory glucose stimulations. Adapted with permission from Zhang et al. (2009) [84]. Copyright © 2009, American Chemical Society

The Piston group was also one of the pioneers to use a microfluidic platform for the study of the pancreatic islet function yielding contrasting findings about the critical role of gap junction coupled K_ATP_ channel activity in controlling insulin secretion. They developed a microfluidic device capable of partially stimulating an islet by trapping and holding it between two separate fluid streams (with different glucose concentrations), revealing that β-cells are effectively coupled to coordinate an intracellular Ca^2+^ response only within regions of the islet in contact with a glucose concentration above a threshold [79] (Fig. 5A). They further examined the behavior of individual cells within an intact islet, using a system to hold the islet stationary in a precise location while maintaining an open path for a continuous fluid flow [80, 81]. These approaches permitted time-lapse imaging of similar regions of the islet after different treatments and shed light on the synchronous glucose-dependent bursts of the electrical activity of the β-cells, demonstrating that, in intact islets, β-cells are coupled by gap junctions, which synchronize electrical activity and intracellular Ca^2+^ oscillations after exposure to stimulatory glucose levels (>7mM for mouse pancreatic islets). However, if glucose concentration is beneath that threshold, β-cells are not sufficiently coupled to spread Ca^2+^ responses into these regions.

Different approaches emerged to exploit high-throughput analysis at the single islet level. Ismagilov and co-workers fabricated a microfluidic “chemistrode” for stimulating, recording, and analyzing molecular signals from a single murine islet. This device consisted of a V-designed tube with an opening point at the vertices of the V. With this design, a solution could be delivered to the substrate by one arm and removed from the other arm after the delivery of a sequence of multiple molecular signals at the substrate-chemistrode interface with pulses as short as 50 ms [82] (Fig. 5B). A novel microfluidic device aimed at quantifying zinc secretion appeared 1 year later with a similar temporal resolution. Zn^2+^ ions, which are cosecreted with insulin from β-cells and therefore could serve as a positive indicator of the secretory capacity of these cells, were quantitatively measured by storing in real-time the islet secretions into droplets, allowing the observation of rapid Zn^2+^ oscillations using a fluorescent indicator [83].

In parallel, a simple method to create temporal gradients of glucose concentrations using two on-chip diaphragm pumps was applied to monitor intracellular calcium levels of a single islet of Langerhans. Following the same scheme, the cell chamber of the chip presented a window below that facilitated fluorescent monitoring of Ca^2+^ changes, showing that intracellular calcium oscillations were modified and regulated by the glucose waves [84, 85] (Fig. 5C). The ability to monitor Ca^2+^ oscillation was also implemented to a microfluidic device able to deliver glucose to a restricted area of a single β-cell, confirming how glucose induces a shift in the spatial distribution of insulin granules within the subcellular portion in contact with the glucose flux. These experiments shed light on the intracellular changes undergone by pancreatic islets under different environments and clearly illustrated the feasibility of using microfluidics coupled with optical systems to study single islet metabolism [86].

A simplified fabricated pumpless microfluidic array driven by surface tension allowed determining real-time fluorescent imaging of [Ca^2+^] without the need of using external pumps to facilitate the delivery of the fluid, which probably made it more applicable for most of the laboratories involved in diabetes research. This system entailed several advantages over conventional microfluidic systems driven with pumps: (i) it required extremely small volume of solutions, therefore achieving a higher analytical spatiotemporal resolution; (ii) it simplified the manageability; and (iii) it avoided bubble generation. Additionally, it allowed the analysis of individual islets from an islet array [87].

A more suited automated perfusion method was developed to investigate the entrainment of insulin secretion from islets in response to glucose waveforms. Of note, insulin secretion from a pancreatic islet is pulsatile, even in response to constant glucose [18, 88]. However, if islets are pooled together in a single chamber with constant glucose, these pulses overlap, resulting in the register of elevated but constant levels of insulin [78]. Periodic variations in glucose can entrain islets and insulin secretion, promoting inter-islet synchronization. With a robust microfluidic system capable of generating different glucose fluctuations to a single islet or a group of islets without dispersion of the waveform, which is significantly difficult to obtain using static techniques, islets could be entrained to generate synchronous insulin pulses in response to oscillatory glucose levels as occurs in vivo [89, 90].

Rapid measurements of changes in insulin levels from a single islet were also possible with a microfluidic device with on-line fluorescent anisotropy immunoassay. The principle of this method was based on the use of fluorescent anisotropy, which measures the depolarization of the fluorescent-labeled insulin versus the free insulin after being excited with linearly polarized light, therefore determining the average anisotropy of the solution. This system demonstrated a very sensitive limit of detection, exhibiting great capability for measuring insulin secretion from a single islet [91]. Similarly, more recently, a fully integrated IOC design using fluorescent anisotropy immunoassay allowed measuring insulin secretion from human islets trapped in parallel. The design of this specific platform was modular and the chip fabrication scalable; therefore, the number of traps on the chip could be altered to offer functional testing using different numbers of islets [92].

The establishment of fluorescence imaging of islets associated with a microfluidic device has also permitted investigating the metabolic control of pancreatic islets exposed to external cues. For instance, it allowed examining the effect of fibroblast growth factor-21 (FGF-21), a potent metabolic regulator [93] that enhances β-cell function and survival on pancreatic islets [94]. To this aim, a unique combination of two-photon and confocal autofluorescence imaging of NAD(P)H and mitochondrial NADH responses in living islets in a microfluidic chip was employed. This enabled gaining mechanistic insights into changes in the mitochondrial metabolism of pancreatic islets exposed to high glucose and palmitate, being both hallmarks of T2D and obesity. The study revealed that FGF-21-treated islets did not show palmitate-induced potentiation of glucose-stimulated mitochondrial activation and insulin secretion. Of interest, β-cells are extremely susceptible to oxidative stress due to high endogenous production of reactive oxygen species (ROS) and low expression levels of antioxidative enzymes [25, 26]. For this reason, a reduced stimulation of mitochondrial responses under metabolic stress could be recognized as a strategy to reduce ROS production, contributing to a longer-term survival of islets. Although accumulating evidence suggests that fat storage in tissues contributes to the development and progression of T2D, a recently discovered new class of branched fatty acid esters of hydroxyl fatty acids (FAHFAs) has been described as positive regulators of glucose metabolism, exhibiting anti-diabetic and anti-inflammatory properties [95, 96], results that were further questioned by Pflimlin and colleagues [97]. However, in parallel, a microfluidic devices was developed to enable quantitative measurements of insulin release dynamics from single islets in response to FAHFAs, reporting enhanced secretion of insulin in response to this lipid [98]. These observations were in line with the first experiments that indicated that FAHFAs presented significant anti-diabetic effects. Nevertheless, further studies are necessary to better comprehend their mechanism of action.

Given the electric characteristics of the islet response together with the need for measuring islet functionality in a rapid, simple, cost-effective, and non-invasive way motivated the development of a dielectric impedance spectroscopy. This technique provided a method able to measure in real-time the cell membrane capacitance and cytoplasmic conductivity, which could be related to stimulus response and functionality assessments. Together with microfluidics, this method offered a tool to detect changes on the dielectric spectra. These alterations correlated with changes in the islet membrane resulting from the fusion of insulin vesicles during secretion in response to dynamic flow, hence offering potential to track islet stimuli response [99].

One of the major challenges of the early platforms was the impossibility to precisely evaluate an individual islet among an entire population, which hindered the assessment of the heterogeneous properties of these pseudo-organs. With this intention, an optimized microfluidic chip, also coupled with real-time microscopy, provided more comprehensive and spatiotemporal analysis of hundreds of islets individually at different time points under different stimulations. This increased the experimental throughput and offered the possibility to identify potential sub-phenotypes among the entire islet population [100].

Recently, an open microfluidic chip with hanging-drop technology that precisely controls fluid enabled the study of time-resolved insulin secretion from single-human islet microtissues [101]. This strategy implied a first step of generating uniform islet spheroids from isolated native human islets, providing control over the size, the endocrine cell composition, and the purity of the sample, and therefore minimizing the inherent heterogeneity of pancreatic islets. With this approach they were able to demonstrate that it is possible to obtain physiological relevant outcomes and corroborated that single islet perfusion enables detection of oscillatory pulsations that are lost when islets are pooled together, probably as a consequence of averaging of asynchronous insulin secretion patterns of the individual islets, as was already suggested before using microfluids [89, 90]. This approach constitutes a robust, simple, and operative tool to recapitulate physiological relevant responses of single islets to glucose and provides the resolution and reproducibility to study novel mechanisms or pharmacological compounds using a small number of replicates.

### Label-free detection of insulin secretion

Despite all the advantages of the microfluidics for the study of pancreatic islet physiology, there is a lack of integration with sensing modules capable of directly monitoring insulin secretion, the key physiological event that is usually perturbed in T2D. Previously reported microfluidic perfusion systems developed to study islet functionality by detecting insulin rely on on-line labeling of this hormone by means of immunofluorescent procedures or off-line quantification of the secreted insulin by ELISA. However, to completely exploit the potential of these platforms, there is a need to interface them with an integrated sensing module capable to monitor directly and labelled-free the islet insulin response.

Our group have developed for the first time an IOC device coupled with a localized surface plasmon resonance (LSPR) sensing module to monitor on-line and label-free the insulin secretion in pancreatic islets [102]. These optical biosensors present the advantage of being highly sensitive, enabling on-line, label-free, and cost-effective sensing and have shown great capability to detect all kinds of molecular biomarkers [103–105]. Unlike other IOC devices, based on multiple tiny wells to trap the islets [65, 68, 70, 106], we have developed a 3D heterogeneous porous polymeric cryogel scaffold to spatially organize the islets. Indeed, we have recently demonstrated that these macroporous scaffold can be used to generate 3D functional β-cell clusters from rat insulin-producing INS1E cells, representing a suitable model to study pancreatic islet function in vitro [107]. Briefly, the biomimetic islet scaffold is subjected to a microfluidic flow that allows the transfer of different glucose concentrations to the chamber of the islets and helps the delivery of the secreted insulin from the IOC to the on-chip LSPR sensing platform. The integration of both platforms allows a highly sensitive and label-free monitoring of on-line insulin secretion by pancreatic islets under physiological conditions, offering a powerful tool for the future biomedicine and pharmaceutical research related with diabetes.

### Long-term studies

In the field of diabetes, it is of interest to develop an in vitro platform setup with the ability to culture islets for long periods. However, most of these studies have used microfluidic devices with short-term applications, mostly as live cell imaging platforms that hold islets stationary while recording multiple real-time physiological changes in response to different treatments. Only few microfluidics platforms have been designed to explore the potential of using islet-on-a-chip devices for long-term purposes. Long-term experiments using microfluidic platforms have been limited in part due to the formation and accumulation of air bubbles in the device. In this sense, Wang et al. proposed a microfluidic platform with an integrated modular bubble trap, demonstrating that a long-term bubble-free system was feasible using a conventional laboratory vacuum for long-term dynamic culture of pancreatic islets [108].

Another issue to take into account when culturing pancreatic islets for long periods is that islet blood vessels are disrupted during the islet isolation process and that static ex vivo culture of islets promotes the loss of the remaining endothelial cells (ECs) [109]. Importantly, pancreatic islets are highly vascularized micro-organs with a dense network of capillaries which facilitate a rapid exchange of nutrients, oxygen, and hormones between endocrine cells and the bloodstream [110]. Hence, it has been suggested that the disruption of the islet vasculature may be the underlying cause explaining why in vitro cultures are unable to sustain primary islets longer than a few days.

Several studies have attempted to examine the integration of islets with microvascular networks [111, 112], showing that vascularized islets significantly enhance survival of diabetic mice after transplantation [113]. The first microfluidic platform aimed at demonstrating that fluidic flow improves maintenance of endothelial cells (ECs) on islet evidenced preserved EC density and reduced necrosis in the core of the islets when exposed to a microfluidic system, indicating that flow rate enhances survival of the β-cells. Additionally, under flow conditions, islets displayed enhanced glucose stimulated Ca^2+^ response, not only demonstrating the feasibility of using microfluidic chips for long-term cell cultures but also suggesting an optimized strategy to increase EC survival in culture prior to islet transplantation, with probably better outcomes [114]. Recently, a microchip-based engineering of uniform-sized spheroids reaggregated from dispersed rat islets under perfusion system pointed to the same direction. Flow not only enhanced the islet health but also maintained the islet endothelial cells (iECs) in vitro over a month, facilitating diffusion-mediated interactions within the islet. On the contrary, under static conditions, the endothelial cells were lost over time [115].

Overall, these results indicate that perfusion systems could offer a means to sustain islets for longer periods, allowing the identification of factors that may contribute to improved β-cell function over time.

## Multi-organ-on-a-chip: seeking new T2D models?

**Fig. 6:**
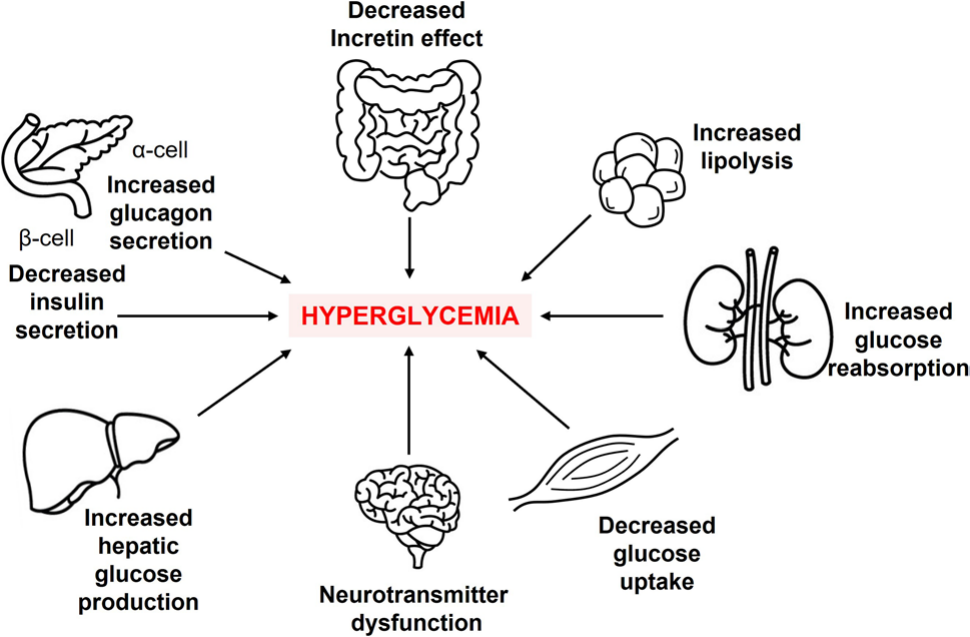
Different organs and tissues involved in glucose homeostasis and multiple defects associated with T2D.

Disorders affecting the metabolic system are often caused by the interaction of multiple factors, involving the crosstalk between different organs and tissues, or even different cellular compartments into the same tissue (Fig. 6). Many physiological stressors can disturb the tightly regulated mechanism of the β-cell function in the context of metabolic overload and insulin resistance commonly found in human obesity-linked T2D [116]. However, there is still a need to further explore the possibilities that these platforms offer for the study of diseases that progress through the disruption of the homeostatic crosstalk between two or more organs as occurs in T2D. Conventional in vitro models to study organ interactions have been limited to co-culturing different cell types and transferring media from cells to cells, failing to recapitulate the time-dependent dynamics of multiorgan interactions. Therefore, to recreate their relevant characteristics in vitro, models with adequate complexity are required.

Organ-on-a-chip platforms represent the perfect candidate tool for the study of multifactorial pathologies such as diabetes, allowing the interplay of different organs and tissues and emulating systemic interactions through on-chip co-cultures of different organs in separate compartments interconnected through microfluidic channels [47, 49]. Such systems were first used to replicate in vivo pharmacokinetic and pharmacodynamic responses of a specific drug [117, 118]. The concept expanded beyond drug metabolism and successive improvements were established to study cell–cell interactions with the creation of 3D environments and long-term cultures [119, 120].

Few approaches have been described so far interconnecting pancreatic islets with other cell types, evidencing the complexity to monitor complex interactions between different tissues when involving pancreatic islets. One of the first microfluidic two-organ-on-a-chip including β-cells was developed to study the pancreatic islet and liver crosstalk [48]. In the liver, insulin stimulates glycogen synthesis and suppresses hepatic glucose production, but signals to stop glycogenolysis and gluconeogenesis. As an anabolic hormone, insulin also promotes protein and lipid synthesis and release from this organ [20].

Based on insulin and glucose regulation, a functional coupling of human pancreatic islet microtissue with liver spheroids composed of primary human stellate cells and HepaRG cells and was developed. To demonstrate its efficacy, pancreatic islets were stimulated with high glucose concentrations, which promoted insulin secretion from β-cells and a concomitant glucose uptake by the liver spheroids. The development of this peristaltic on-chip micropump enabled a continuous pulsatile flow, promoting high tissue perfusion rates and allowing a long-term co-culture up to 15 days. Interestingly, islets in co-culture with liver spheroids maintained the secretory capacity of the β-cells to secrete insulin in response to glucose along the total duration of the experiment; however, islet microtissues cultured alone presented decreased GSIS over time. This enhanced islet functionality has been also recently demonstrated with the development of a new pancreas-liver-on-chip [121]. Functional assays revealed that the crosstalk between primary rat hepatocytes and pancreatic islets under flow conditions resulted in enhanced insulin and C-peptide secretion from islets.

The secretory capacity of the β-cells can be also modulated by other factors. Obesity itself is a risk factor for diabetes as it causes the elevation of several proinflammatory cytokines and chemokines that are released by adipocytes and are thought to contribute to the inflammatory process in several tissues including the liver and pancreatic islets [122–124]. Apart from inflammatory intermediates, adipocytes secrete nonesterified fatty acids (NEFAs) and adipokines that can also have an impact on the β-cell function [125, 126]. A simple and effective chip design permitted exposure of adipocyte secretions to a single islet through perfusion and monitoring of glucose-stimulated insulin secretion in real-time with an integrated electrophoresis-based immunoassay [127]. Short-term exposure of islets to adipocyte perfusion augmented insulin secretion, demonstrating that acute exposure to adipocyte-released factors can enhance insulin secretion. Of note, lipid signaling is necessary for GSIS and the rapid effect of NEFAs potentiating GSIS has been widely demonstrated in vitro, suggesting that they act to amplify the coupling mechanisms of the metabolic stimulus and the secretion [128]. However, future studies should be directed towards employing organ-on-chip technology to address the long-term effects of these factors released by the adipose tissue on pancreatic islets.

The muscle tissue is also a main player in the development of T2D as, in the postprandial state, skeletal muscle is the predominant site of insulin-mediated glucose uptake. In the muscle tissue, insulin resistance, defined as a complex pathological state in which insulin-dependent cells fail to respond to insulin, is considered to be a primary defect of T2D, occurring before β-cell failure [19, 129]. Indeed, the skeletal muscle itself is considered an endocrine organ that secretes a variety of molecules which can regulate the metabolic function in other tissues and organs [130, 131]. Indeed, muscle-secreted factors have already been proven to influence β-cell function [132, 133] and given that release of skeletal muscle-derived molecules is enhanced by muscle contraction, a platform to interrogate the skeletal muscle and β-cell crosstalk is required to understand and the beneficial effects of exercise associated with β-cell function in patients with prediabetes and T2D [134]. For this reason, bioengineered technologies allowing the formation of aligned myofibers are emerging as new tools for preclinical research [135].

To better comprehend the interaction between skeletal muscle and β-cells, a perfusion platform examined real-time effects of molecules released by contracting C2C12 skeletal muscle myotubes on insulin release by INS1 pseudoislets and human islets. Conditioned media from stimulated myotubes with electric pulses influenced positively insulin secretion in β-cells from non-diabetic and T2D patients and increased the mitochondrial respiratory activity of β-cells [136].

A step forward for personalized medicine using organ-on-chip technology was presented recently with the development of a unique pancreatic function monitoring tool to study cystic fibrosis (CF)-related disorders in vitro [137]. CF is a genetic condition affecting the gene encoding the cystic fibrosis transmembrane conductance regulator (CFTR), localized on the epithelial cells in multiple organs, including the pancreas [138]. In CF patients, cell–cell signaling between pancreatic ductal epithelial cells (PDECs) and islet cells is disturbed, which consequently affects the secretory capacity of the β-cells [139, 140]. Indeed, subjects with CF have an increasing risk of developing diabetes [141].

The development of a pancreas-on-a-chip allowed mimicry of the functional interface between PDECs and pancreatic islets while monitoring cell–cell functional interaction directly and efficiently using only a small number of patient-derived cells, demonstrating that ductal cells and islets are functionally coupled and corroborating that CFTR plays a relevant role in directly regulating insulin secretion. This patient-derived in vitro model opens the door to personalized medicine as it can be set up for multiple cell types and several analyses.

## Conclusions

Several islets-on-a-chip platforms have been designed to combine specific tissue microenvironments and architectures within a microfabricated device that can exhibit functional hallmarks of the native pancreatic islets. These new platforms have emerged as complex in vitro models to study the islet physiology, representing a new way to study β-cell function and failure with high resolution.

However, despite all the improvements to create advanced fabricated platforms, most of the islet-on-chip assays have heavily relied on fluorescence microscopy and have mainly been used for specialized proof-of-concept studies to investigate insulin secretion. There is still a need to design more sophisticated islet-on-chip platforms equipped with built-in sensors that provide valuable real-time data on a cellular level, which cannot be easily measured in animal models. These new approaches may provide comprehensive 3D in vitro solutions for investigating metabolic disease pathophysiology and screening new drugs and therapies for T2D.
